# A Case of Critical Aortic Stenosis Masquerading as Acute Coronary Syndrome

**DOI:** 10.4061/2010/423465

**Published:** 2010-07-20

**Authors:** Siddharth A. Wayangankar, Tarun W. Dasari, Pedro M. Lozano, Karen J. Beckman

**Affiliations:** ^1^Department Internal Medicine, University of Oklahoma Health Sciences Center, 920 Stanton L. Young Blvd., WP 1130, Oklahoma City, OK 73104-5020, USA; ^2^Cardiovascular Sciences Section, University of Oklahoma Health Sciences Center, Oklahoma City, USA

## Abstract

Serum cardiac troponins I and T are reliable and highly specific markers of myocardial injury. Studies have shown that at least 20% of patients with severe aortic stenosis have detectable serum troponins. This case report describes a patient who presented as suspected acute coronary syndrome with markedly elevated troponin levels, who was later found to have normal coronaries and critical aortic stenosis. This case highlights the need for comprehensive and accurate physical examination in patients who present with angina. Critical aortic stenosis may cause such severe subendocardial ischemia as to cause marked elevation in cardiac markers and mimic an acute coronary syndrome. Careful physical examination will lead to an earlier use of non invasive techniques, such as echocardiography to confirm the correct diagnosis and the avoidance of inappropriate treatments such as intravenous nitroglycerin and glycoprotein IIb/IIIa inhibitors.

## 1. Introduction

In patients with aortic valve stenosis, the development of left ventricular systolic dysfunction and heart failure predicts poor prognosis, including a less favorable outcome after valve replacement [1, 2]. The onset of heart failure is preceded by structural and functional alterations in the heart muscle with left ventricular hypertrophy followed by degeneration and death of the cardiac myocytes [3]. Serum cardiac troponins I and T are heart specific contraction-regulating proteins released into the circulation from injured myocytes. Their value in acute coronary syndrome is well known and they are assuming a growing prognostic role in heart failure as well [4–9]. Critical aortic stenosis (defined as valve area <0.5 mm^2^) has been reported to cause modest elevations in cardiac biomarkers such as troponin I [10].

We present an unusual case of critical aortic stenosis that masqueraded as acute coronary syndrome until definitive studies ruled out the latter.

## 2. Case Presentation

**Figure 1:**
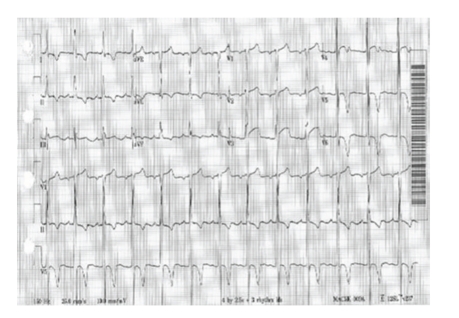
Electrocardiogram showing left ventricle hypertrophy and repolarization abnormality but no acute ST-T changes suggestive of myocardial injury.

**Figure 2:**
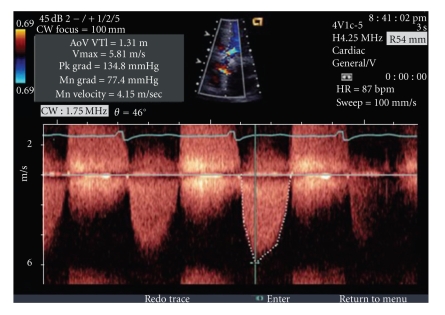
Echocardiogram showing criteria for critical aortic stenosis.

A forty-nine-year-old otherwise healthy Caucasian male with history of marijuana abuse presented with 12 hours of constant retrosternal, nonradiating chest pain, partially relieved by sublingual nitroglycerine. Physical exam was significant for BP 140/74, heart rate 75 beats/min, carotid shudder, single S2, S4 gallop, and a grade IV/VI crescendo-decrescendo late-peaking murmur in aortic area that radiated to the carotids. These physical examination signs were consistent with critical aortic stenosis. Laboratory work revealed a troponin rise from an initial level of 0.09 ng/mL (normal: <0.4 ng/mL), CK-MB of 3.7 ng/mL (normal: 0–11.2 ng/mL)), to a second troponin value of 26.7 ng/mL which finally peaked at 99 ng/mL (CK-MB of 81.3 ng/mL) within 12–18 hours of presentation. Urine Drug Screen was negative for cocaine but positive for marijuana. A 12-lead EKG revealed sinus rhythm at 75 beats/min with left ventricle hypertrophy and repolarization abnormality (Figure 1) but no acute ST-T changes suggestive of myocardial injury. The classic physical exam findings of aortic stenosis lead us to an urgent echocardiography, rather than an early invasive approach of cardiac catheterization. Echocardiography revealed (Figure 2) an ejection fraction of 55–60%, suspected bicuspid aortic valve with critical aortic stenosis and peak velocity of 5.8 m/sec, a peak transvalvular gradient of 135 mm of Hg (mean gradient of 77 mm of Hg), and an indexed valve area of 0.52  cm^2^, all of which were suggestive of critical aortic stenosis. 

Elective coronary angiography done a day later revealed normal coronaries. The aortic valve could not be crossed. The patient underwent uncomplicated aortic valve replacement surgery and is doing well. 

## 3. Discussion

**Figure 3:**
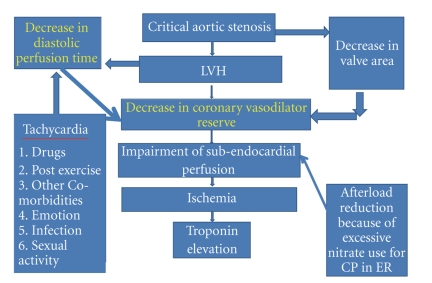
Proposed pathophysiology of troponinemia in our patient with critical aortic stenosis.

Severe aortic stenosis usually causes compensatory left ventricular hypertrophy. The ventricular hypertrophy may result in a reduction in coronary vasodilator reserve (CVR), which is the ratio of maximal to basal coronary blood flow. CVR can be used as a functional index of the severity of coronary artery insufficiency despite angiographically normal coronary arteries [11]. A similar reduction in CVR has also been observed in patients with hypertrophic cardiomyopathy and hypertension [12]. In these patients, the diastolic perfusion time (DPT) has a significant effect on CVR: the faster the heart rate, the lower the CVR [13]. The combination of left ventricular hypertrophy, tachycardia, and lower perfusion pressure (which can occur in critical aortic stenosis) will significantly impair subendocardial perfusion resulting in ischemia (Figure 3). Rajappan et al. demonstrated that there was no obvious relationship between the degree of hypertrophy and CVR whereas on the other hand the interaction of DPT and valve area seemed to be the critical parameter in the development of ischemia in aortic stenosis [14]. 

Circulating cardiac troponin I (cTnI) is detectable in more than one-half and elevated in more than one-fifth of patients with severe aortic stenosis [15, 16]. Importantly, cTnI is elevated even in the absence of systolic dysfunction indicating that it can precede overt LV dysfunction. As per Nunes et al., serial monitoring of cTnI could help clinicians identify the start of the inevitable clinical downhill in severe aortic stenosis [16]. Whether such high levels of troponins secondary to severe subendocardial ischemia portend poor prognosis warrants further investigation. 

The usefulness of an accurate and thorough clinical exam in the emergency department is of paramount importance in such patients. Administration of nitrates in the emergency room might have actually worsened this patient's subendocardial ischemia. The combination of decreased end diastolic volume (preload) along with decreased afterload with nitrate use may have further compromised the effective stroke volume and thus ischemia. 

We were cognizant of the fact that this patient had a positive urine test for cannabis. Based on the history, patient last consumed marijuana three weeks prior to admission. Though there have been few case reports of cannabis-induced myocardial damage, these cases usually were associated with recent use of cannabis. On the basis of a case-crossover analysis that controlled for differences between patients, Mittleman et al. found that within 1 hour after smoking marijuana, the risk of myocardial infarction onset was elevated 4.8-fold (95% confidence interval, 2.9–9.5; *P* < .001). In the second hour after smoking marijuana, the relative risk was 1.7 (95% confidence interval, 0.6–5.1; *P* = .34), suggesting a rapid decline in the cardiac effects of marijuana [17]. The risk of ischemic events is linked to tachycardia and hypertension secondary to excess sympathetic surge associated with cannabis. However our patient had heart rate between 70 and 80 and systolic blood pressures between 140 and 150 mm of Hg at admission. Moreover, the cannabis metabolites persist in urine for as many as 77 days post abstinence and hence should not be used as an index of acute ingestion [18].

## 4. Conclusion

Extreme elevations in cardiac troponins are rare in the absence of acute coronary syndromes. This is an unusual case of critical aortic stenosis presenting as acute coronary syndrome and highlights the importance of a thorough physical exam and the early use of noninvasive testing such as echocardiography. The underlying mechanisms of such extreme elevation in cardiac biomarkers in patients with critical aortic stenosis are unclear but could be related to decreased coronary vasodilator reserve and diastolic perfusion time. The use of nitrates in the initial triage for angina may worsen the subendocardial ischemia in this subset of population and hence should be used with caution. Finally, since ischemia in this setting is an index of microcirculatory dysfunction, research is needed to determine whether the absolute value of troponins correlates with an increased predisposition to systolic dysfunction and a worse prognosis in patients with aortic stenosis.
